# Changes of Constituents and Activity to Apoptosis and Cell Cycle During Fermentation of Tea

**DOI:** 10.3390/ijms12031862

**Published:** 2011-03-10

**Authors:** Hang Zhao, Min Zhang, Lu Zhao, Ya-kun Ge, Jun Sheng, Wei Shi

**Affiliations:** 1 Key Laboratory for Molecular Enzymology & Engineering, the Ministry of Education, Jilin University, Changchun 130012, China; E-Mails: zhzky@163.com (H.Z.); zhangmin2584652@yahoo.com.cn (M.Z.); zhaolu_1987@126.com (L.Z.); yakunge@126.com (Y.-K.G.); 2 College of Life Science, Jilin University, Changchun 130012, China; 3 Yunnan Research Centre for Advance Tea Processing, Yunnan Agricultural University, Kunming 650201, China

**Keywords:** tea, catechins, theabrownins, caffeine, apoptosis, cell cycle

## Abstract

Tea is believed to be beneficial for health, and the effects of the fermentation process on its contributions to apoptosis and cell cycle arrest of gastric cancer cells have not been completely investigated. In this study, the chemical components in green tea, black tea and pu-erh tea aqueous extracts were analyzed and compared. The polysaccharide and caffeine levels were substantially higher in the fermented black tea and pu-erh tea, while the polyphenol level was higher in the unfermented green tea. Hence, a treatment of tea aqueous extract and the components, which are emerging as promising anticancer agents, were pursued to determine whether this treatment could lead to enhance apoptosis and cell cycle arrest. In the human gastric cancer cell line SGC-7901, the cell viability and flow cytometry analysis for apoptotic cells indicated effects in a dose-dependent inhibition manner for the three tea treatment groups. The apoptosis rates were found to be elevated after 48 h of treatment with 31.2, 125, and 500 μg/mL of green tea extract, the higher catechins content may be involved in the mechanism. Cell cycle was arrested in S phase in the fermented black tea and pu-erh tea, and the populations were significantly decreased in G2/M phases, possibly due to the oxidation of tea polyphenols, which causes an increase of theabrownins. CCC-HEL-1 normal cells were not sensitive to tea extract. These findings suggest that the fermentation process causes changes of the compounds which might be involved in the changes of cell proliferation inhibition, apoptosis induction and cell cycle arrest.

## 1. Introduction

Tea is one of the most popular and widely consumed beverages in the world because of its refreshing taste, attractive aroma, and its possible beneficial health effects that are being extensively investigated and have received a great deal of attention in recent times [1–4]. Generally, tea can be broadly classified according to the production method as unfermented tea (green tea), fully fermented tea (black tea) and post-fermented tea (pu-erh tea) [5,6]. Nowadays, a lot of *in vitro* studies, animal and human research have demonstrated biological functions of tea, such as anti-bacterial, anti-viral, anti-oxidation, high potential of protection against atherosclerosis and cardiovascular diseases [7,8]. These beneficial effects have been attributed to the presence of tea compounds such as catechins, polysaccharides, theabrownins and caffeine. Content and composition of the constituents vary substantially among the various teas, depending on the degree of fermentation and on the individual mode of preparation [9].

**Figure 1.**
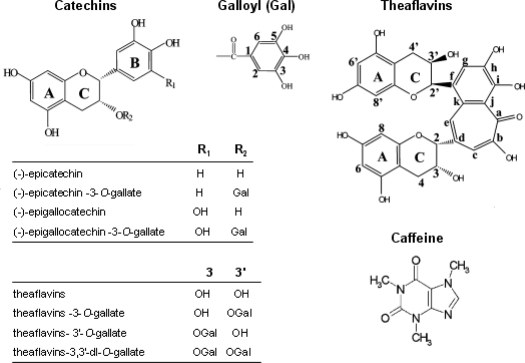
Chemical structures of the investigated compounds in tea [23].

Catechins, which comprise epigallocatechin-3-gallate (EGCG), epigallocatechin (EGC), epicatechin-3-gallate (ECG), and epicatechin (EC), are members of the four main tea phenolic compounds. Catechins have attracted significant attention recently [10]. The manufacturing process is designed to either prevent or allow tea polyphenols to be oxidized by naturally occurring polyphenol oxidase enzymes in the leaves. The production of green tea is to avoid the oxidation of polyphenols. In contrast, black tea and pu-erh tea are produced by promoting enzymatic oxidation of tea polyphenols. During the fermentation process, catechins are oxidized to complex compounds. Theaflavins, thearubigins and theabrownins are the main pigments and complex phenolic compounds deriving from the oxidation of catechins and their gallates during this processing. Theaflavins undergo further oxidation during fermentation to form more polymerized thearubigins, and then condensed theabrownins [11–13]. Caffeine exists widely in the leaves, seeds and fruits of a large number of plants. It is obtained by extraction from tea or coffee, fermentation of dry tea enhances or reduces its caffeine content mainly due to the molds. The change of caffeine with fermentation time is similar in the fermentation process [14,15]. The chemical structures of some compounds are illustrated in Figure 1.

The possible cancer preventive activity of tea has received much attention in recent years. The inhibitory activities of tea and tea constituents against carcinogenesis have been demonstrated in many animal models [16–18]. Gastric cancer is of major importance world-wide, being the second most common cause of cancer-related death in the world [19]. Since some of the treatments that induce apoptosis are cell cycle specific and all of them in some way will disrupt the cell cycle, an investigation of the relationship between the cell cycle and apoptosis can be of great value. The cell cycle phase from which apoptosis has been triggered can be directly measured. After some treatments, cells may progress through the cell cycle before undergoing apoptosis, in which case a different set of techniques will need to be employed [20–22]. In this study, we select three kinds of tea aqueous extract produced in Yunnan, China. Analysis of the content of the constituents in them and the effect related to apoptosis and cell cycle in gastric cancer SGC-7901 cells and CCC-HEL-1 normal cells is reported.

## 2. Results

### 2.1. Chemical Structures of the Investigated Compounds

### 2.2. Contents of Several Polyphenol Ingredients in Teas

Because the samples of tea extract have been obtained from the same locations, the origins and manufacturing processes of these tea samples are similar. Therefore, we decided to study the effect of the fermentation process on the levels of these constituents. In this study, the fermentation processes are carried out by tea-making experts in the China Academy of Pu-erh Tea Research. The resulting tea products are classified according to the degree of fermentation as unfermented tea (green tea), fully fermented tea (black tea) and post-fermented tea (pu-erh tea). The active ingredients, including tea polyphenol, polysaccharides and caffeine in green tea, black tea and pu-erh tea were analyzed and compared in our study (Table 1). It seems that the degree of fermentation has a profound effect on the levels of polyphenol. Total polyphenol levels in tea aqueous were significantly decreased from 56.23% to 33.13% during fermentation. In addition, polysaccharide levels increased during the fermentation process. The caffeine content of the three tea samples ranged from 8.62% to 9.31% (w/w) of the extract. It did not change much depending on the degree of fermentation. The biochemical mechanism of this elevation is interesting and deserves further investigation. The tendency of changes via the fermentation agreed with previous research [24,25]. Tea pigments are due to the oxidation of tea catechins and their derivatives. Tea pigment levels were substantially increased in the fermented tea [11]. Generally tea pigments consist of theaflavins, thearubigins, and theabrownins. Theabrownins are the most stable component with the largest molecular weight [26].

### 2.3. Inhibitory Effect on the Proliferation of SGC-7901 Cells

**Figure 2.**
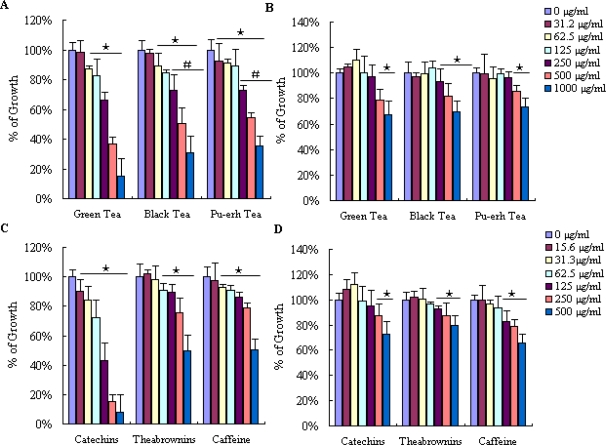
Proliferation of SGC-7901 and CCC-HEL-1 cells exposed to various drugs for 48 h by MTT assay. The proliferation rates of (**A**) SGC-7901 and (**B**) CCC-HEL-1 cells treated with three tea extract at the concentrations of 31.2–1000 μg/mL. The proliferation rates of (**C**) SGC-7901 and (**D**) CCC-HEL-1 cells treated with three main compounds present in tea extract at the concentrations of 15.6–500 μg/mL (as indicated concentration). ★ p < 0.05 when compared with that of the positive control group (only treated with DMEM). # p < 0.05 when compared with that of the green tea cell group at the same concentration.

The human gastric cancer cells SGC-7901 and CCC-HEL-1 normal cells were treated with various concentrations of tea extract and their main compounds for 48 h, which induced a significant decrease in MTT reduction. The cell viability was expressed as MTT conversion rate. In SGC-7901 cells, green tea, black tea and pu-erh tea extract could inhibit the growth of gastric cancer cells in a dose dependent manner (Figure 2A). There was not much difference depending on the concentration of 31.2, 62.5 and 125 μg/mL. Cell viability was decreased to 66.4%, 36.5% and 15% with green tea extract at 250, 500 and 1000 μg/mL, respectively. In contrast, black and pu-erh tea extract produced a slight increase in cell viability compared with green tea. In the black tea extract treatment group the cell viability was 73.0%, 50.7% and 31%. While with pu-erh tea extract treatment, the result was 75.2%, 54.6% and 35.2%. The 50%-inhibition concentrations (IC_50_) value of SGC-7901 cells at 48 h was 335.9, 511.5, 658.1 μg/mL, respectively. The cell viability of the two groups that underwent fermentation process was relatively higher than the green tea treatment. It was noted that green tea had more inhibitory effect than black tea and pu-erh tea on cell growth.

The IC_50_ value of catechins, theabrownins and caffeine on SGC-7901 cells at 48 h was 99.1, 522.0, 808.5 μg/mL, respectively. Samples at the concentration of 15.6–500 μg/mL on cell viability was depicted (Figure 2C). Treated with theabrownins at concentrations of 15.6–62.5 μg/ml showed no significantly difference, but catechins treatment decreased cell viability by 10–30% at the concentration in this experiment. Treatment of tea constituents at different concentrations for 48 h decreased cell viability and suppressed about 50–90% relative to the untreated cell group at 500 μg/mL. It appeared that catechins showed greater effects than theabrownins and caffeine. Various authors have reported anticancer effects of green tea catechins. In those studies, catechins showed anticancer effects by decreasing cell viability and increasing caspase-3 activity in many cells [27].

The *in vitro* cytotoxicity in SGC-7901 cells has been attributed, thus its interaction in CCC-HEL-1 normal cells were compared. The IC_50_ values for the three tea extract were more than 1000 μg/mL (Figure 2B), and for catechins and theabrownins were more than 500 μg/mL (Figure 2D). Relatively higher cell viability was found from each group compared to SCG-7901 cells, but no statistical differences were noted for the caffeine treatment of the two cell types. The studies presented herein showed that tea extract and their constituents were more cytotoxic to carcinoma SGC-7901 cells than the normal CCC-HEL-1 cells.

### 2.4. Flow Cytometric Analysis of Cell Apoptosis

**Figure 3.**
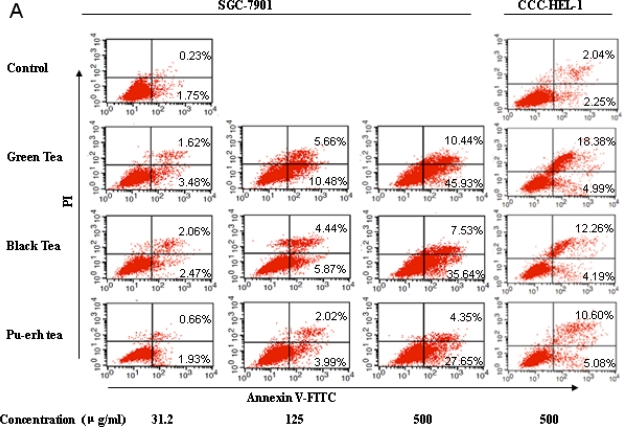

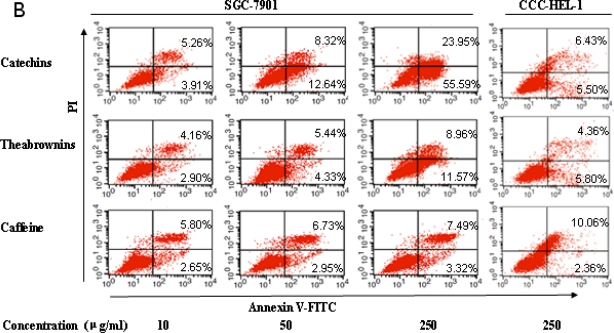
Flow cytometric analysis of cell apoptosis induced by treatment for 48 h in SGC-7901 and CCC-HEL-1 cells. (**A**) Apoptotic cells after treatment with three tea extracts at concentrations of 31.2, 125 and 500 μg/mL in SCG-7901 cells and 500 μg/mL in CCC-HEL-1 cells; (**B**) Apoptotic cells after treatment with tea constituents at the concentrations of 10, 50 and 250 μg/mL in SCG-7901 cells and 250 μg/mL in CCC-HEL-1 cells.

To investigate whether apoptosis contributed to cell growth inhibition by tea extracts and their components, an assessment of apoptosis rate was observed using flow cytometry. Both the early stage of apoptosis (lower-right) and the late stage of apoptosis (upper-right) in SGC-7901 and CCC-HEL-1 cells were investigated. We examined the concentration-dependence of the samples on apoptosis in SGC-7901 cells. As demonstrated, treatment with tea extracts for 48 h induced early and late stage apoptosis (Figure 3A). Results showed that the percentage of early apoptosis increased to 3.48 ± 0.42%, 2.47 ± 0.45% and 1.93 ± 0.79% compared with 1.75 ± 0.36% in control cells by 31.2 μg/mL green tea, black tea and pu-erh tea, respectively. Treatment with 500 μg/mL increased the percentage of early apoptosis to 45.93 ± 3.25%, 35.64 ± 3.17 and 27.65 ± 2.19%, respectively. The data indicated that the three kinds of tea extract could induce early apoptosis, and the percentage induced in the green tea extract groups was higher than the black tea group, with the lowest rate in pu-erh tea groups at the same concentration .The fermentation process causes changes of the compounds, which may result in reducing the apoptosis of SGC-7901 cells. Early apoptosis was induced by the addition of catechins in the SGC-7901 cell line, rising from 3.91 ± 0.32% to 55.59 ± 5.02% (Figure 3B). The combination of catechins also increased late apoptosis, but insignificantly greater than early apoptosis. A significant reduction in early apoptotic activity was observed with theabrownins when compared to the catechins at the same concentration. Late apoptosis was significantly observed by the addition of caffeine, and no significant changes in late apoptosis were observed with different concentrations.

The results from apoptotic analysis for the CCC-HEL-1 normal cells showed that the percentage of early apoptosis after treatment 500 μg/mL green tea, black tea and pu-erh tea was 4.99 ± 0.98%, 4.19 ± 0.36% and 5.08 ± 0.73%. 250 μg/mL catechins and theabrownins induced slight apoptosis (right side of Figure 3A and B). The percentage of apoptosis was significantly lower than observed in SGC-7901 cells at the same concentration. The data indicated that tea extract and constituents induced early apoptosis in the carcinoma cells, but normal cells were not sensitive, which may play a role in the *in vivo* inhibition of tumorigenesis.

### 2.5. Flow Cytometric Analysis of Cell Cycle

**Figure 4.**
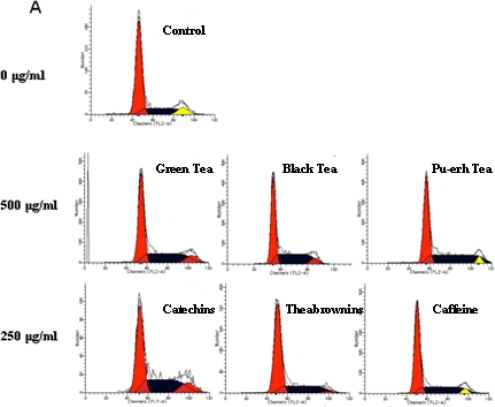

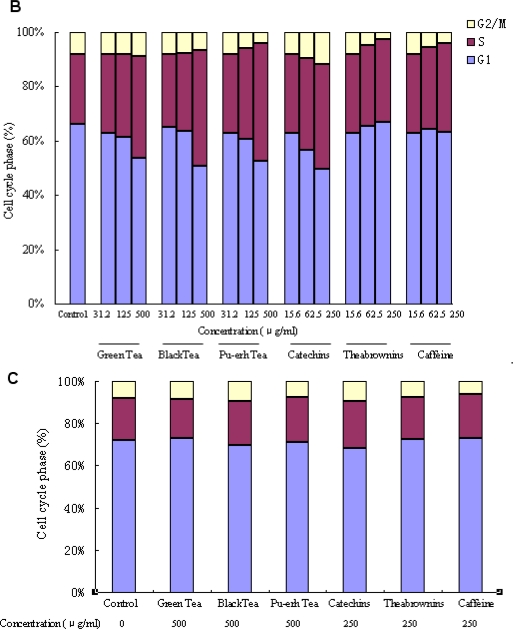
Cell cycle analysis of SGC-7901 and CCC-HEL-1 cells after treatment with three kinds of tea extract and their main constituents. (**A**) Cell cycle phase distributions of SGC-7901 cells cultured under three same concentrations with apoptosis analysis, and the high concentration of each tea extract and the constituents were shown; (**B**) The data with various concentration treatments in SGC-7901 cells was calculated and expressed by histogram; (**C**) Cell cycle phase distributions of CCC-HEL-1 cells cultured under the same concentration with apoptosis, and the data was calculated and expressed by histogram.

The tea extract and tea main compounds-mediated inhibition of cell proliferation was then examined by investigating the effects on cell-cycle distribution after treatment with three concentrations (Figure 4B). Cell populations in the G1, S and G2/M phases were 66.19%, 25.81% and 8.00%, respectively, in control SGC-7901 cells. After 48 h of incubation with 500 μg/mL green, black and pu-erh tea extract, the population of S phase cells increased to 37.49%, 42.83% and 43.67%, the population of G2/M phase cells in the green tea groups was almost identical to that in the population of control cells, while the populations were significantly decreased to 6.59% and 3.59% in black and pu-erh tea groups (Figure 4A). The result indicated that the fermentation process may be involved in the changes.

To assess the activity of the tea constituents in SGC-7901 cells, cells were treated with three constituents at the indicated concentration. The cell cycle distribution of the cells is shown in Figure 4A. The catechins yielded a cell population with 38% and 11.66% of the cells at the S and G2/M boundary. Theabrownins resulted in 30.30% and 2.43% of the cells, respectively. In the presence of theabrownins, the population at the G2/M boundary was 30% of that of the non-treated cells. The value increased progressively through G2/M to S phases during the fermentation process. However, the effect of caffeine did not vary significantly. The percentage of cells in the different stages of the cell cycle with various concentration treatments was further analyzed by histogram (Figure 4B). For the CCC-HEL-1 normal cells, no statistical differences were noted for each group (Figure 4C).

## 3. Experimental Section

### 3.1. Materials

Dulbecco’s modified Eagle medium (DMEM), newborn calf serum, and 3-(4, 5-dimethylthiazol-2-yl)- 2, 5-diphenyltetrazoliunbromide (MTT) were purchased from GIBCO BRL (Grand Island, NY, USA).

Trypsin, penicillin, streptomycin and all other chemicals employed in this study were of analytical grade and were purchased from Sigma Chemical Co. (St. Louis, USA). Catechins, theabrownins and caffeine were provided by China Academy of Pu-erh Tea Research (Pu Erh, Yunnan, China). Fluorescein isothiocyanate-conjugated annexin V (Annexin V-FITC) and propidium iodide (PI) Apoptosis Detection Kits was purchased from BD Biosciences (Pharmingen, USA). Propidium iodide (PI) for cell cycle analysis was from Calbiochem (La Jolla, Canada).

### 3.2. Preparation of Tea Extracts

The tea leaves of green tea, black tea and pu-erh tea were collected from plants grown in the Yunnan Highlands of China. Green tea leaves were collected and heated, dried at <60 °C and molded to make unfermented tea. To make fermented black tea and post fermented pu-erh tea, the tea leaves were dampened and fermented, then dried at <60 °C and packed. Green tea, black tea and pu-erh tea were extracted three times by placing in boiling distilled water for 10 min each time. The solution was collected, lyophilized to obtain the aqueous extract.

### 3.3. Determination of Polyphenol, Polysaccharides, and Caffeine Content in Concentrated Tea Extracts

Determination of polyphenol content was performed under the guidelines of national standards using the ferrous tartrate method [11,28]. Briefly, the tea extraction solution, buffer solution and ferrous tartrate tetrahydrate solution were mixed in a 25 mL capacity bottle. Absorbance (A) at 540 nm with a 10 mm quartz cell was used to calculate the extraction of tea polyphenols. Polysaccharides were quantitated using the anthrone–sulfuric acid method using glucose as standard as described [29]. A standard curve was generated with glucan, which was linear between the concentration range of 5 and 30 μg. The calibration curve equation was y = 0.063x + 0.0579 and had a correlation coefficient of R^2^ = 0.9957. Caffeine was quantitated using the lead subacetate method [11]. A standard curve was generated with caffeine, which was linear in the concentration range of 50 and 300 μg. The calibration curve equation was y = 62.911x + 0.0058 and had a correlation coefficient of R^2^ = 0.9997.

### 3.4. Cell Culture and Cell Proliferation Assay

Catechins, theabrownins, caffeine, green tea, black tea and pu-erh tea extract were dissolved in complete DMEM, the pH value adjusted to 7.2 and sterilized through a 0.2 μm filter to the desired working solutions (equivalent to 15.6–1000 μg/mL, w/v). Human gastric cancer cell line SGC-7901 was provided by the Cell Bank of Shanghai Institute of Cell Biology, Chinese Academy of Sciences (Shanghai, China). Human primary embryo liver-derived cells CCC-HEL-1 was obtained from cell center of the Chinese Academy of Medical Sciences and Peking Union Medical College. Cells were cultured in DMEM medium supplemented with 10% fetal bovine serum (FBS), 100 mg/mL streptomycin and 100 units/mL penicillin at 37 °C in a humidified incubator in an atmosphere of 5% CO_2_.

SGC-7901 and CCC-HEL-1 cells were seeded in 96-well plates (1 × 10^4^ cells/well) for 24 h incubation, cell viability was evaluated using MTT assay as described previously [30]. In brief, cells were treated with green tea, black tea, pu-erh tea, catechins, theabrownins and caffeine at a various concentration for 48 h and untreated cells served as a control. Prior to determination, 5 μL MTT (2.5 g/L) was added to each well. After 4 h incubation, the culture media were discarded followed by addition of 100 μL of DMSO to each well and vibration for 10 min. The absorbance (A) in the experimental wells was measured at 570 nm with a microplate reader. The absorbance in the experimental wells to that of the control wells (without test compound). The percentage of viable cells was calculated as follows: (A of experimental group/A of control group) × 100%. Following this, the IC_50_ (cytotoxic concentration for 50% cell death) was determined from the dose-response curve.

### 3.5. Flow Cytometry Analysis of Apoptosis, Cell Cycle in SGC-7901 Cells

SGC-7901 and CCC-HEL-1 cells were seeded in 6-well plates (4 × 10^5^ cells/well) for 24 h incubation and treated with various drugs at the indicated concentrations for 48 h. Apoptosis of cells was evaluated by measuring the exposure of phosphatidylserine on the cell membranes using Apoptosis Detection Kits. Cell pellets were resuspended in a staining solution containing PI and Annexin V-FITC for 15 min at room temperature in the dark. The cells were assessed by FACS equipped with the Cell Quest software (BD, Pharmingen). Cell cycle analysis was undertaken by flow cytometric analysis after propidium iodide (PI) staining. Briefly, cells in suspension with and without drugs were fixed with ethanol at 4 °C for 24 h and then stained with 50 μg/mL of PI, 100 μg/mL RNase A in a PBS solution. After staining, the population of cells in each cell cycle phase was determined using the ModFit software (BD, USA).

### 3.6. Statistical Analyses

All tests and chemical determinations were made in at least triplicate, and the data are expressed as means ± standard error of mean (S.E.M.). Statistical analyses were evaluated using Student’s t-test. Analysis of variances and pairwise comparisons were examined by ANOVA single-factor test at the P < 0.05 confidence level.

## 4. Discussion

Although the anti-carcinogenic activities of tea have been demonstrated in many studies, epidemiological evidence for a protective role of tea consumption against cancer in human populations is weak. These inconsistencies may be due to the insufficient intake of tea. Therefore, to understand the chemopreventive effect of tea, higher amounts may have to be consumed. A second possible reason for the discrepancy between human epidemiological studies and experiments is that the human population is not homogenous in genetic makeup and life style, and the results are influenced by many confounding factors [31,32]. Some researchers believe that the digestive tract, which can have direct contact with tea constituents, holds greater promise to prevent cancer. Some results are very exciting, the dose of 200 mg green tea polyphenols three times a day did not produce significant side or adverse effects and should stimulate many similar cancer prevention studies [31].

The use of tea, as a cancer chemopreventive agent has been appreciated in the last twenty years. It has now been suggested that tea polyphenols potently induce apoptotic cell death and cell cycle arrest in tumor cells but not in their normal cell counterparts and affect several biological pathways. As supporting evidence, various animal studies have revealed that treatment with tea inhibits tumor incidence and multiplicity in different organ sites such as skin, lung, liver, stomach, mammary gland, and colon [33,34]. Several studies comparing the *in vitro* cytotoxicity of EGCG have shown its greater toxicity to cancer than to normal cells and have suggested that EGCG, the most abundant polyphenolic in green tea, was the prime agent mediating the chemopreventive properties of green tea [35]. Such findings, coupled with *in vivo* studies showing green and black tea extracts inhibit tumorigenesis in animal model systems, are suggestive of the potential protective role of teas against human cancers [36]. The cancerous cells were more susceptible to cytotoxicity induced by the polyphenol of tea than the normal cells [34,37].

## 5. Conclusions

The data acquired from chemical components and flow cytometric analysis in this study gave us clues to the key molecules that contribute to apoptosis and cell cycle in gastric cancer. We found that catechins, the main tea phenolic compounds, could induce early apoptosis of gastric cancer cell lines SGC-7901 higher, and during the fermentation process the content of phenolic compounds reduced. In addition, cell cycle results showed that the proportion of G2/M phase cells decreased with the fermentation process, which may be due to the oxidation of tea polyphenols, and increase of theabrownins. In addition, our investigation showed that tea extract and their constituents have lower cytotoxicity to normal CCC-HEL-1 cells. Induction of early apoptosis occurred only in SGC-7901 cancer cells, but not in CCC-HEL-1 normal cells. Cell cycle was not affected with the high concentration treatment in this study. Although further studies are required to elucidate the molecular mechanisms, these results suggest that tea extract and their constituents could be a candidate agent for the therapy of gastric cancer.

## Figures and Tables

**Table 1.** Contents of several ingredients in teas (w/w).

**Sample**	**Polyphenols**	**Polysaccharid**	**Caffeine**	**Theabrownins**
Green Tea	56.23 ± 5.17	1.01 ± 0.11	8.62 ± 0.14	---
Black Tea	42.40 ± 3.35	3.42 ± 0.05	8.92 ± 0.19	---
Pu-erh Tea	33.13 ± 3.18	4.81 ± 0.13	9.31 ± 0.09	7.32–10.50
